# The Smoking Paradox in Stroke Patients Under Reperfusion Treatment Is Associated With Endothelial Dysfunction

**DOI:** 10.3389/fneur.2022.841484

**Published:** 2022-03-24

**Authors:** Ramón Iglesias-Rey, Antía Custodia, Maria Luz Alonso-Alonso, Iria López-Dequidt, Manuel Rodríguez-Yáñez, José M. Pumar, José Castillo, Tomás Sobrino, Francisco Campos, Andres da Silva-Candal, Pablo Hervella

**Affiliations:** ^1^Neuroimaging and Biotechnology Laboratory, Health Research Institute of Santiago de Compostela, Santiago de Compostela, Spain; ^2^Clinical Neurosciences Research Laboratories, Health Research Institute of Santiago de Compostela, Santiago de Compostela, Spain; ^3^NeuroAging Group, Health Research Institute of Santiago de Compostela (IDIS), Santiago de Compostela, Spain; ^4^Stroke Unit, Department of Neurology, Hospital Clínico Universitario, Santiago de Compostela, Santiago de Compostela, Spain; ^5^Department of Neuroradiology, Hospital Clínico Universitario de Santiago de Compostela, Universidade de Santiago de Compostela, Santiago de Compostela, Spain; ^6^Neurovascular Diseases Laboratory, Neurology Service, University Hospital Complex of A Coruña, Biomedical Research Institute, A Coruña, Spain

**Keywords:** stroke, reperfusion, leukoaraiosis, sTWEAK, endothelial dysfunction

## Abstract

**Objective:**

This study aimed to explore the association between smoking habit and the serum levels of soluble tumor necrosis factor-like weak inducer of apoptosis (sTWEAK), in relation with the functional outcome of patients with acute ischemic stroke undergoing reperfusion treatment.

**Methods:**

Observational and retrospective study of a series of patients with acute ischemic stroke subjected to reperfusion treatments. Clinical, analytical, and neuroimaging parameters were analyzed. The main endpoint was the functional outcome at 3 months, measured by the modified Ranking Scale (mRS). Logistic regression models were used to analyze the association between smoking and sTWEAK levels with functional outcome and leukoaraiosis.

**Results:**

The results showed that smoking habit was associated with a good functional outcome at 3 months in patients with stroke (OR: 3.52; 95% CI: 1.03–11.9; *p* = 0.044). However, this independent association was lost after adjusting by sTWEAK levels (OR 1.73; 95% CI: 0.86–13.28; *p* = 0.116). sTWEAK levels were significantly lower in smoker patients [4015.5 (973.66–7921.83) pg/ml vs. 5,628 (2,848–10,202) pg/ml, *p* < 0.0001], while sTWEAK levels were significantly higher in patients with poor functional outcomes at 3 months [10,284 (7,388–13.247) pg/ml vs. 3,405 (2,329–6,629) pg/ml, *p* < 0.0001].

**Conclusion:**

The decrease in sTWEAK levels was associated with a good functional outcome in smoker patients with stroke undergoing reperfusion therapy.

## Introduction

Recent clinical studies in cerebrovascular diseases have shown unexpected associations of stroke progression with demographic and clinical data suggesting a better outcome for men (1), obese people (2, 3), and smokers (4) among patients with stroke. Moreover, these associations are even more pronounced in patients undergoing reperfusion treatments (5–7), although those studies are inconclusive and should be questioned.

The controversial beneficial effect of the smoking habit in patients with stroke has been associated with a lower number of complications and good functional outcome (4, 5, 8–10) and could be partially explained by the well-known increased fibrinolytic activity (11) in smoker patients, although there are other disagreeing studies on these associations (12, 13). Therefore, the actual mechanisms behind the positive neurovascular effect of tobacco remain unclear. Several possible mechanisms have been postulated, such as the tobacco effect over the blood-brain barrier (BBB), angiogenesis and increased capillary density, stimulation of growth factors, oxidative stress, or increased metabolic activity (14, 15), but all these hypotheses still need to be confirmed.

In this study, we hypothesize that smoking habit could enhance the thrombolytic activity partially through the activation of the tumor necrosis factor-like weak inducer of apoptosis-fibroblast growth factor-inducible molecule 14 (TWEAK-Fn14) pathway. This hypothesis is based on common neuroinflammatory mechanisms shared by the soluble TWEAK (sTWEAK) and tobacco, as well as on the similar action over the BBB integrity (16). Moreover, sTWEAK and tobacco share a possible common participation in phenomena associated with ischemic preconditioning and on the activation of the NF-κB (nuclear factor kappa-light-chain-enhancer of activated B cells) (14, 15, 17–19).

Based on the paradoxical effect of tobacco over ischemic stroke, the objectives of our study were as follows: 1) to determine the influence of smoking habit on the outcome in a series of patients with acute ischemic stroke undergoing reperfusion treatments; 2) to study the possible association between tobacco consumption and sTWEAK levels measured at the time of admission of patients with acute ischemic stroke undergoing reperfusion treatment.

## Methods

### Ethics Approval

This study was carried out in accordance with the Declaration of Helsinki of the World Medical Association (2008) and was approved by the Clinical Research Ethics Committee of Galicia (registration codes 2019/616 and 2016/399). Informed consent was obtained from patients or their relatives at the time of inclusion in the registry, authorizing the anonymous use of data for further studies.

### Study Design and Protocols

This is an observational and retrospective study of a series of patients with acute ischemic stroke included consecutively and prospectively in a data bank (BICHUS). The following inclusion criteria were previously defined as follows: 1) authorization for the anonymous use of patient data for research; 2) no history of previous ischemic or hemorrhagic stroke; 3) an MRI or CT study at admission and between the 4th day and the 7th day; 4) patients undergoing some kind of reperfusion treatment; 5) known time of the onset of stroke, with a latency time between the onset and administration of the reperfusion procedure in less than 6 h; 6) face-to-face or telephone monitoring at 3 months ± 15 days; and 7) blood sample stored in the biobank. Only 27 patients who died within the first 24 h were excluded.

All patients with smoking habits, at least during the last year, were considered smokers regardless of their intensity. On the other hand, patients who have never smoked or patients who were abstinent at least during the last year were identified as non-smokers. The stroke was classified according to the Trial of ORG 10172 in Acute Stroke Treatment (TOAST) criteria (20). The intensity of the neurological deficit was determined by the National Institute of Health Stroke Scale (NIHSS) upon admission in the stroke unit and every 6 h during their stay in the unit. We consider early neurological deterioration with an increase of ≥ 4 points in the NIHSS in the first 48 h. The functional deficit was assessed by the modified Ranking Scale (mRS) at 3 months ± 15 days (face-to-face in 80.8% of the sample). Functional impairment at 3 months was categorized as a good outcome if the mRS ≤ 2 and a poor outcome if the mRS > 2. Both scales were evaluated by internationally certified neurologists. All patients were admitted to the stroke unit and were treated with the protocol of the Spanish Neurological Society, by neurologists trained in cerebrovascular diseases. The recanalization was assessed by the neurological improvement, determined by a decrease of eight points in the NIHSS in the first 24 h.

### Analytical Variables

Blood samples were obtained from all patients at admission and collected in test tubes and centrifuged at 3,000 g for 15 min, and serum was immediately frozen and stored at −80°C. Serum concentrations of sTWEAK were determined in samples obtained at admission, before the administration of the reperfusion treatment. Serum levels of sTWEAK were measured using commercial ELISA kits (Elabsciences) following the manufacturer's instructions. The intra- and inter-assay variation coefficients were 5.1 and 5.2%, respectively. All determinations were performed in a laboratory blinded to clinical data. Other laboratory tests were conducted by the Laboratory of the University Clinical Hospital of Santiago de Compostela and were performed on fresh blood samples at the time of diagnosis or shortly after.

### Neuroimaging Studies

The identification of the symptomatic hemorrhagic transformation (European Cooperative Acute Stroke Study (ECASS) criteria) (21) was performed at the time of recording the neurological worsening and, in any case, in a second CT performed between the 4th day and the 7th day. The volume of the lesion was determined in the CT performed between the 4th day and the 7th day. The volumes were determined using the ABC/2 method until 2016 and later through the automated planimetric method. The images of leukoaraiosis were stratified according to the Fazekas scale (22) and were identified in the neuroimaging performed at admission. All the neuroimaging studies were supervised by the same neuroradiologist.

### Statistical Method

The number of patients was calculated in the base of sTWEAK determinations to achieve a power of 80% to detect differences in the contrast of the hypothesis null H_0_ (the difference in means is equal to the limit of superiority, by means of a unilateral *t*-student test of superiority for two independent samples). Taking into account that the level of significance is 5% and assuming that the upper limit is 20,000 pg/ml, the average of sTWEAK in the group of the good outcome is 2,035.5 pg/ml and of the poor outcome is 4,150 pg/ml. The standard deviation (SD) of both groups is ~500 pg/ml. It will be necessary to include at least 168 individuals with a poor outcome and 392 with a good outcome. In addition, 10 more cases should be included for each adjustment covariate in the multivariate analysis (3–4 of a poor outcome and 6–7 of a good outcome, depending on the proportion justified). Assuming the need to adjust the model for 220 covariables, at least 248 cases with a poor outcome and 497 with a good outcome are needed. The calculation has been made with Ene software version 3.0. For this analysis, we consecutively selected all the patients who met all of the inclusion criteria from December 2018 backward.

For statistical analysis, a descriptive analysis was primarily performed. Categorical variables were described with frequency and percentage and the continuous ones with mean and *SD* or median and interquartile range, depending on their adjustment to a normal distribution (which was determined with the Kolmogorov-Smirnov test with the Lilliefors correction). Then, the statistical inference was carried out with the chi-square test, Student's *t*-student, or the Mann-Whitney test according to the nature of the contrast variable and its adjustment to normality. Finally, multivariate logistic regression was proposed to be adjusted by the significant variables found in the previous analysis. ORs and their 95% CIs were calculated. All these analyses were performed with IBM SPSS 20 (IBM, Armonk, NY, USA). *P* < 0.05 were considered significant.

### Data Availability

All data are available within the text of the manuscript. Further anonymized data could be made available to qualified investigators upon reasonable request.

## Results

We included 875 patients in our study (45.9% women, mean age 72.0 ± 12.5 years), among which 197 patients (22.5%) were identified as smokers (34.0% women). All patients were cigarette smokers, except for 17 patients (11 cigar and 6 pipe smokers). The average time between the onset of symptoms and the administration of reperfusion treatment was 161.8 ± 61.2 min (95% CI: 30–352 min). Regarding the reperfusion treatment, 710 patients received systemic thrombolysis (tPA), 87 had thrombectomy, and 78 had intravenous or intraarterial thrombolysis followed by thrombectomy. According to the TOAST classification, 206 patients had an atherothrombotic infarction, 381 had cardioembolic, 11 had lacunar, and 277 had undetermined origin (among which 143 patients were identified as such due to the coexistence of two causes and 134 patients without a known cause).

### Influence of Smoking Habit on the Functional Outcome

**Figure 1:**
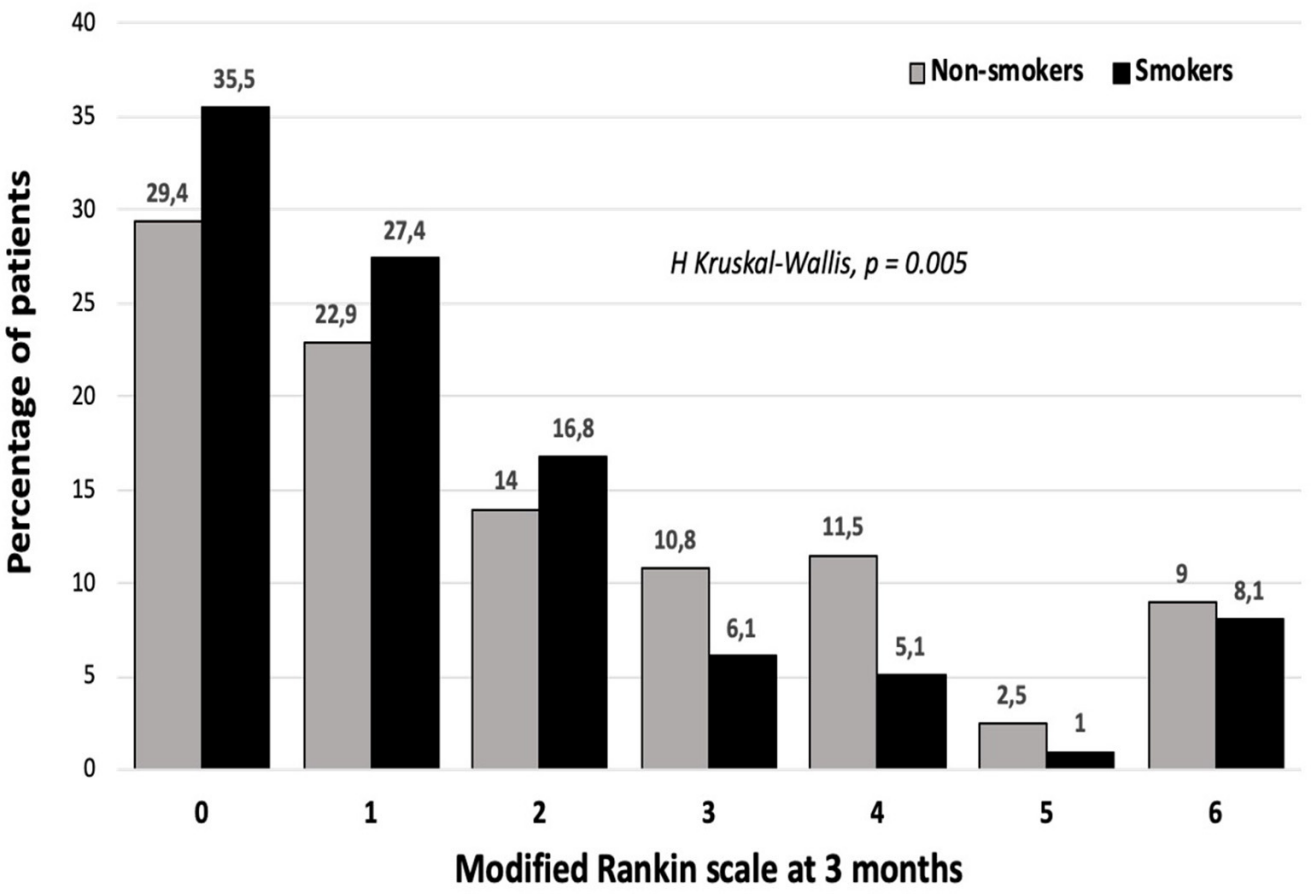
The Ranking scale distribution among smoker and non-smoker patients.

Good functional outcome, defined as a mRS ≤ 2 at 3 months, was observed in 79.7% of the smoker patients, as opposed to in 66.2% of non-smokers patients (*p* < 0.0001); we observed a higher percentage of smoker patients at all mRS ≤ 2 points at 3 months (Figure 1); while non-smoker patients showed higher percentages at mRS > 2. The bivariate analysis for functional outcome at 3 months (Supplementary Table S1) confirmed that the good outcome group included 25.9% of smoker patients, while only 14.9% of smoker patients were observed in the group with poor outcomes (*p* < 0.0001).

In a non-adjusted logistic regression model using the good functional outcome as the dependent variable, we observed an association between smoking habit and good functional outcome at 3 months with an OR of 1.725 (95% CI: 1.175–2.53; *p* = 0.005). This association was maintained after adjusting by the clinical and analytical variables that showed differences between patients with good and poor prognosis (summarized in Supplementary Table S1): age, sex, latency time, temperature at admission, NIHSS at admission, lesion volume in DWI, leukoaraiosis, hemorrhagic transformation and recanalization method (OR: 3.38; 95% CI: 1.004–11.42*; p* = 0.049). However, this association was lost after adjusting by sTWEAK levels (OR 3.07; 95% CI: 0.86–13.28; *p* = 0.085), while sTWEAK levels showed an inverse association with good outcomes in the abovementioned model (sTWEAK per 100 pg/ml: OR 0.87; 95% CI: 0.8–0.95*; p* < 0.0001).

### Mechanisms Associated With Chronic Smoking Habit

The analysis of the clinical and analytical variables of patients classified as smokers and non-smokers is shown in Table 1. The average age was 6 years lower in smokers while the percentage of women was lower in smoker patients. The percentages of patients with hypertensive, diabetic, and atrial fibrillation were lower in the smoker group. Regarding the analytical variables, the fibrinogen levels in smoker patients were lower than that in the non-smoker group, although rest of the inflammation markers (leukocytes and C-reactive protein) were similar. The NIHSS at admission was similar in the two groups; however, effective reperfusion was more frequent in smoker patients.

**Table 1:** Clinical variables, biochemical parameters, and neuroimaging values of patients classified according to their smoking habit.

	**Non-smokers**	**Smokers**	***p***
	***n* = 678**	***n* = 197**	
Age, years	73.3 ± 11.9	67.6 ± 13.2	<0.0001
Woman, %	49.4	34.0	<0.0001
Onset-treatment time, min	163.4 ± 62.3	156.5 ± 56.9	0.161
Previous mRS	0 [0, 0]	0 [0, 0]	0.056
Arterial hypertension, %	66.6	53.3	0.006
Diabetes, %	24.6	16.8	0.021
Alcohol abuse, %	6.8	22.3	<0.0001
Dyslipidemia, %	39.8	37.6	0.619
Peripheral arterial disease, %	6.3	8.1	0.419
Atrial fibrillation, %	25.5	13.7	<0.0001
Ischemic heart disease, %	11.8	15.7	0.146
Heart failure, %	4.4	4.1	0.937
Axillary temperature, °C	36.4 ± 0.7	36.3 ± 0.6	0.119
Blood glucose, mg/dl	139.2 ± 55.6	136.8 ± 56.2	0.592
Leucocytes × 10^3^/ml	8.3 ± 3.2	8.3 ± 3.2	0.822
Fibrinogen, mg/dl	425.0 ± 103.1	397.5 ± 90.6	0.002
C-reactive protein, mg/L	4.3 ± 4.4	3.5 ± 3.8	0.054
Glycosylated hemoglobin, %	5.9 ± 1.2	6.9 ± 9.1	0.059
LDL-cholesterol, mg/dl	106.2 ± 41.7	114.1 ± 35.5	0.060
HDL-cholesterol, mg/dl	41.8 ± 14.5	41.3 ± 16.4	0.762
Triglycerides, mg/dl	110.6 ± 48.3	116.4 ± 59.7	0.131
NIHSS at admission	17 [12, 22]	16 [12, 21]	0.289
TOAST			0.053
Atherothrombotic, %	22.3	27.9	
Cardioembolic, %	46.2	34.5	
Lacunar, %	1.0	2.0	
Undetermined, %	30.5	35.5	
DWI volume at admission, ml	27.3 ± 33.3	31.1 ± 68.9	0.561
CT volume 4th-7th day, ml	52.3 ± 76.3	46.8 ± 74.2	0.372
Leukoaraiosis, %	60.3	51.3	0.029
Degree of leukoaraiosis			0.009
No, %	39.7	48.7	
Fazecas I, %	34.4	36.5	
Fazecas II, %	15.2	11.7	
Fazecas III, %	10.7	3.1	
Hemorrhagic transformation, %			0.115
No, %	67.4	70.1	
IH1, %	20.5	24.5	
IH2, %	5.9	3.0	
PH1, %	3.4	1.5	
PH2, %	2.8	1.0	
Neurological improvement ≥ 8, %	47.9	66.0	<0.0001
Early neurological deterioration, %	11.6	5.6	<0.0001
mRS at discharge	3 [2, 4]	2 [1, 4]	0.029
mRS at 3 months	1 [0, 3]	1 [0, 2]	0.005
sTWEAK (pg/ml)	5,628 [2,848–10,202]	4,015 [974–7,922]	<0.0001

In the adjusted logistic regression model (Supplementary Table S2), smoking habit was independently associated with age (OR 0.97; 95% CI:0.96–0.99; *p* < 0.0001), alcohol consumption (OR 3.27; 95% CI: 2.03–5.27, *p* < 0.0001), and fibrinogen levels (OR 0.99; 95% CI: 0.99–0.99; *p* = 0.009).

**Figure 2:**
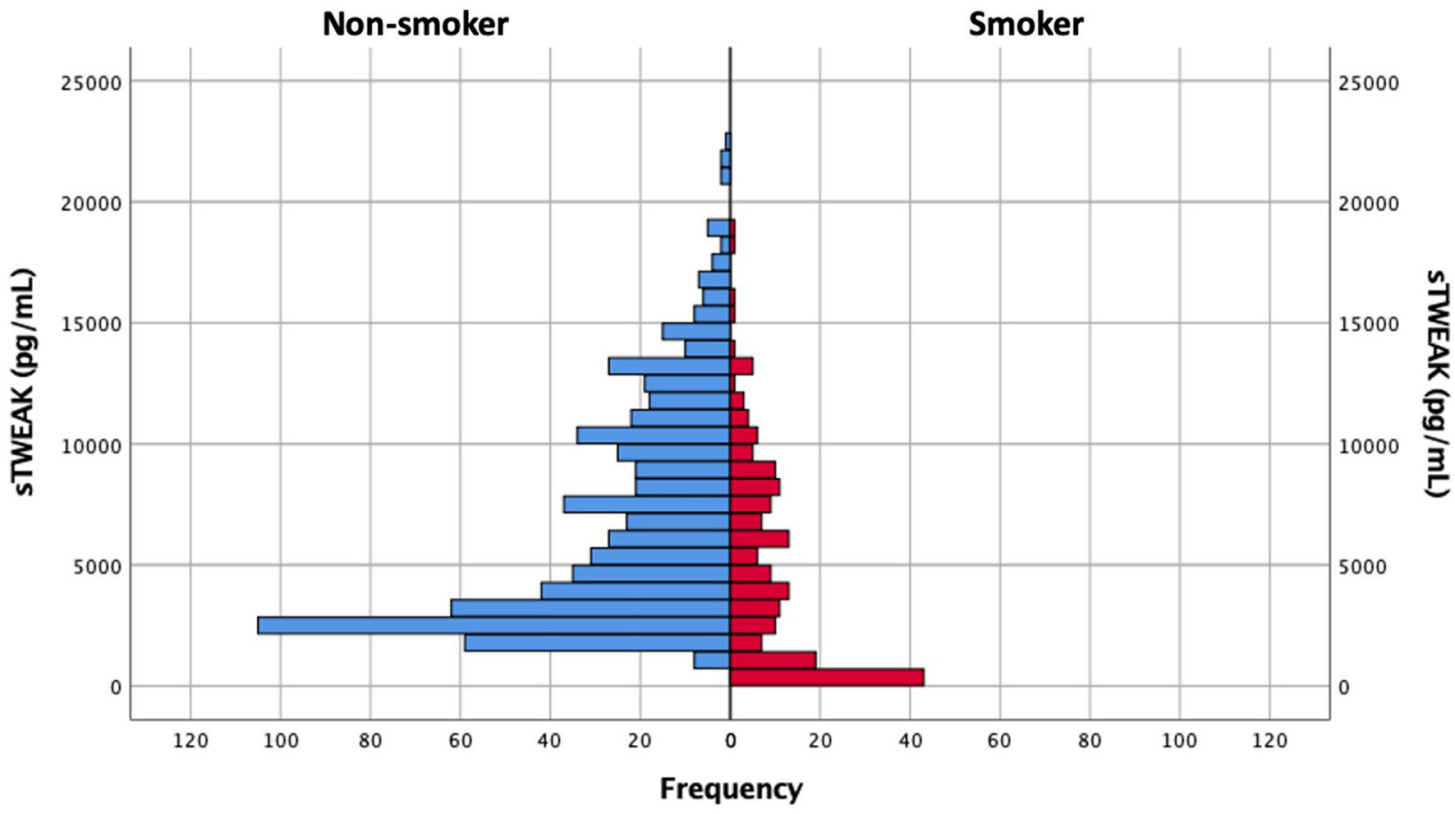
Distribution of soluble tumor necrosis factor-like weak inducer of apoptosis (sTWEAK) values in non-smoker and smoker patient populations.

**Figure 3:**
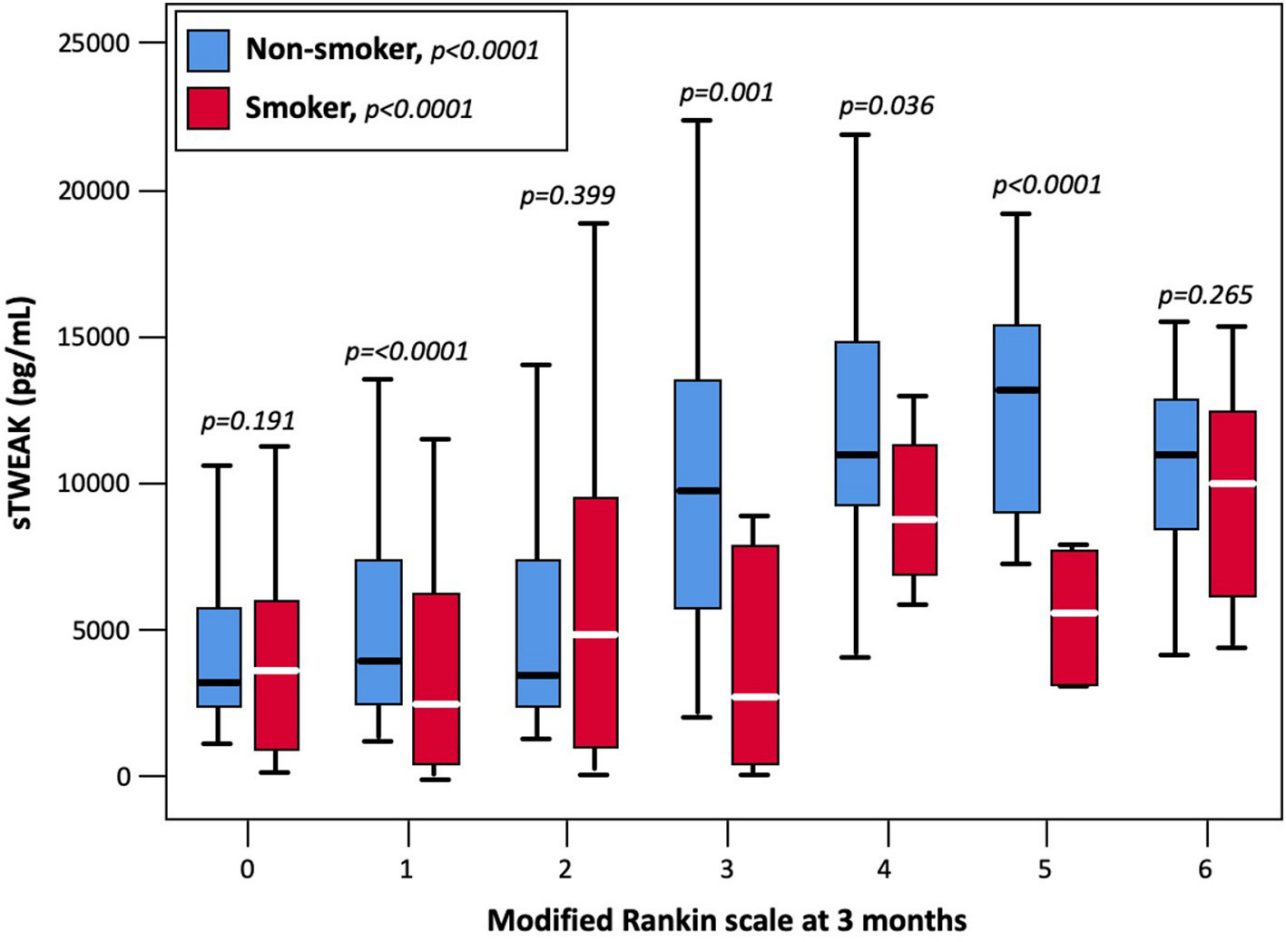
Basal sTWEAK concentrations at different points of the modified Rankin scale at 3 months in smoker and non-smokers patients.

Serum concentrations of sTWEAK were determined in samples obtained at admission before the administration of the reperfusion treatment. sTWEAK levels were significantly lower in smoker patients [4,015 (974–7,922) pg/ml vs. 5,629 (2,848–10,202) pg/ml, *p* < 0.0001] (Figure 2) and were significantly higher in patients with poor outcome at 3 months, compared with patients with good outcomes [10,284 (7,388–13,247) pg/ml vs. 3,405 (2,329–6,629) pg/ml, *p* < 0.0001] (Figure 3). Basal sTWEAK levels were independently associated with smoking habit (OR 0.9: 95% CI: 0.86–0.94; *p* < 0.0001) (Table 2).

**Table 2:** Logistic regression models for factors associated with smoking habit.

**Independent variables**	**Not adjusted**			**Adjusted**		
	**OR**	**CI 95%**	***p***	**OR**	**CI 95%**	***p***
Age	0.97	0.95–0.98	<0.0001	0.97	0.96–0.99	<0.0001
Women	0.53	0.38–0.73	<0.0001	0.79	0.55–1.14	0.217
Hypertension	0.63	0.46–0.87	0.001	0.98	0.67–1.43	0.927
Diabetes	0.62	0.41–0.93	0.021	0.66	0.42–1.03	0.071
Alcohol	3.95	2.52–6.14	<0.0001	3.17	1.95–5.18	<0.0001
Atrial fibrillation	0.46	0.30–0.72	0.001	0.64	0.40–1.03	0.067
Fibrinogen	0.99	0.99–0.99	0.002	0.99	0.99–0.99	0.024
Leukoaraiosis	0.69	0.50–0.95	0.024	1.16	0.80–1.70	0.428
sTWEAK per 100 pg/ml	0.89	0.85–0.93	<0.0001	0.90	0.86–0.94	<0.0001

### Values in Non-smoker and Smoker Patient Populations

**Figure 4:**
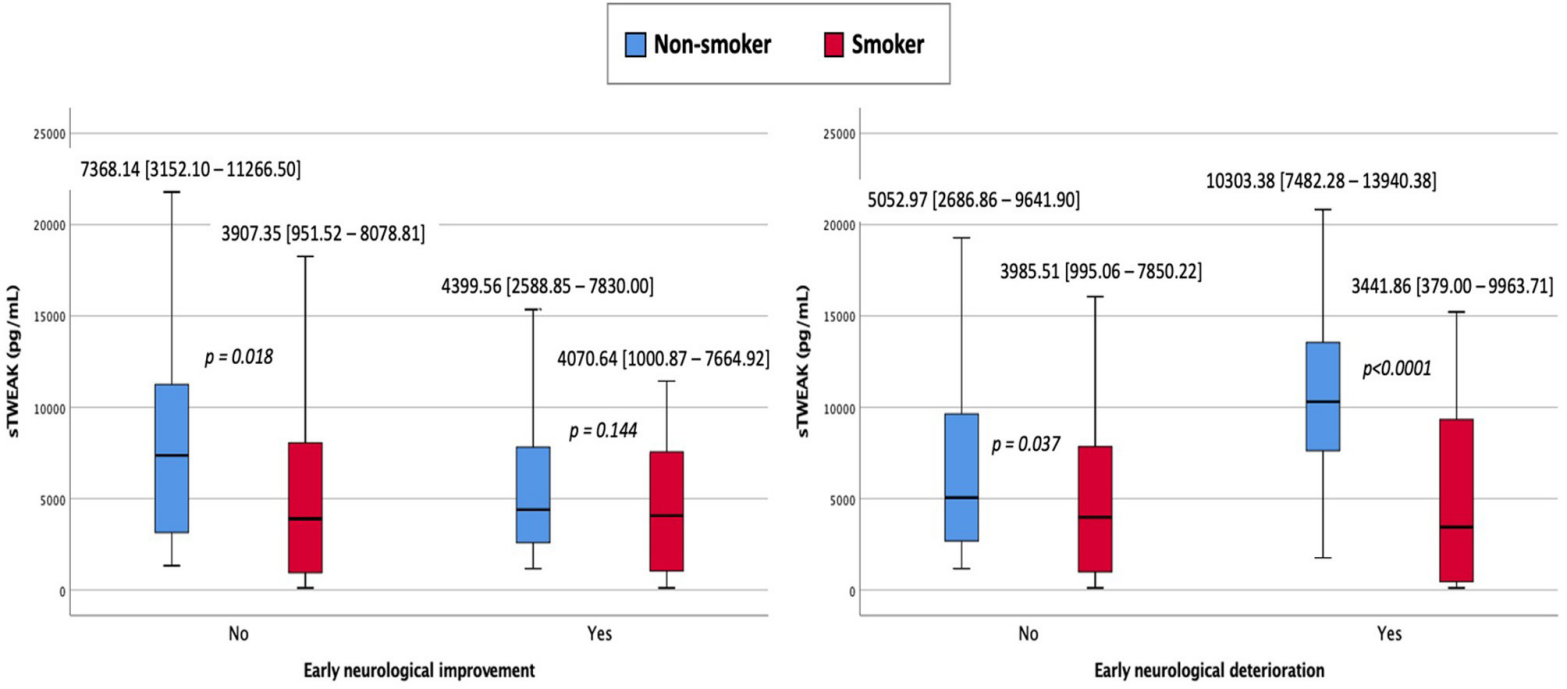
sTWEAK concentrations in smoker and non-smoker patients who presented early neurological improvement and early neurological deterioration.

Smoker patients showed less early neurological deterioration (5.6 vs. 11.6%, *p* < 0.0001) and a higher percentage of early neurological improvement (66 vs. 47.9%, *p* < 0.0001) compared with non-smoking patients. The same relationship was observed for sTWEAK levels that were lower in smoker patients with early neurological improvement [4,234 (2,498–7,785) pg/ml vs. 6,571 (2,923–10,630) pg/ml, *p* < 0.0001] and higher in smokers who presented early neurological deterioration [10,125 (6,435–13,409) pg/ml vs. 4,704 (2,588–8,935) pg/ml, *p* < 0.0001]. The differences observed in sTWEAK levels between smokers and non-smoker patients were especially more pronounced among patients showing early neurological deterioration (Figure 4).

## Discussion

Nowadays, active and passive smoking are associated with the most prevalent causes of mortality and morbidity (23–25). Therefore, smoking suppression and hypertension control are undoubtedly two important objectives that should lead the actions to control preventable deaths (26). Among vascular diseases, the relationship between tobacco and coronary heart disease is well known (27), although its actual mechanism is still unclear (28), and the association of tobacco with stroke is still controversial (27, 29).

In our series of patients with stroke undergoing reperfusion treatment, the association between smoking and good outcome was confirmed. Recent studies suggest the influence of age over the association between stroke and smoke (30, 31). However, we observed in our series an independent association between smoking and good outcomes after adjusting by age.

The influence of tobacco on the fibrinolytic plasma activity remains inconclusive. Some studies showed that nicotine levels are associated with plasminogen activator inhibition (32), while others demonstrated that tobacco increases the fibrinolytic activity (11). In our sample, the clinical response to reperfusion was higher in smoker patients, and we did not observe an increase in the frequency or intensity of hemorrhagic transformations in smoker patients. These discrepancies have been explained by the increased tendency of thrombogenic strokes in smoker patients rather than strokes caused by the rupture of atheromatous plaques with the subsequent formation of platelet-rich clots (4). In this sense, our smoker patients were less cardioembolic and also presented fewer risk factors for atheromatosis and had a greater (non-significant) proportion of stroke of undetermined origin. Another controversial aspect is the association of tobacco with the inflammatory response. The abovementioned hypothesis of tobacco being less associated with the rupture of atheroma plaques (4) is in disagreement with the possibility of a high inflammatory response (33). Our patients had lower concentrations of fibrinogen and C-reactive protein and was also associated with a lower degree of leukoaraiosis (neuroimage associated with inflammatory response) (34, 35).

Most studies analyzing the molecular mechanisms associated with smoking were focused on its effect on the respiratory and cardiovascular systems (14), while the effect of tobacco on the cerebral parenchyma and microcirculation is less known. Some studies have identified effects due to nicotine (36, 37), oxidative stress linked to tobacco (36, 38), and alterations associated with other products derived from tobacco (39). Nicotine exposure could affect the integrity of the blood–brain barrier by causing a decrease in the expression of endothelial tight junction proteins and an increased permeability and edema associated with leukoaraiosis and small vessel disease (40). On the other hand, it was reported that nicotine decreases the activity of Na^+^/K^+^/Cl^2−^ cotransporters through cell membranes (41) and increases the density through a stimulus of angiogenesis (42) and the expression of vascular growth factors (43).

Given the variety of compounds found in tobacco smoke, it is difficult to reach definitive conclusions justifying the smoker's paradox. One of the hypotheses is the ischemic preconditioning, caused by the chronic development of vasoconstriction of small cerebral vessels (4). In *in vitro* models, it was observed that tobacco smoke could actually increase cell death in a culture of cardiomyocytes; however, this effect was lower in cell cultures previously conditioned by oxygen and glucose deprivation (15). Overexpression of p53 is another route leading to cell death (44), which is partially inhibited by exposure to tobacco extracts in hypoxic conditions (15). These studies suggest that tobacco causes cell death in situations of normoxia, but in hypoxic conditions, there is a notable decrease in cell death, a situation that could explain the smoker's paradox.

The benefits of tobacco found in the thrombolysis efficiency are not translatable to clinical practice and under no circumstances should change the current guides regarding the prohibition of tobacco. As an alternative, some studies have focused on the molecular mechanisms behind the benefits of tobacco in acute cerebral ischemia. In this sense, we have evaluated the role of sTWEAK in several mechanisms associated with ischemic brain damage (45). sTWEAK is a part of the TNF superfamily that binds to Fn14 and is expressed in the very early stages of cerebral ischemia, which may explain its association with the early neurological improvement or deterioration. TWEAK-Fn14 induces a neuroinflammatory response, a secretion of proinflammatory cytokines and metalloproteases, and a disruption of the blood-brain barrier (18). Recently, TWEAK-Fn14 signaling has been shown to contribute to the protective effect associated with ischemic preconditioning (18). All these mechanisms are involved in cerebrovascular effects associated with tobacco. Based on these pieces of evidence, we analyzed the sTWEAK levels in patients with reperfused cerebral infarction, since this is a subgroup of patients where the smoker's paradox is clearer. Our results confirmed that the sTWEAK levels were lower in smokers. Similarly, the association between tobacco and good outcome disappeared after introducing the sTWEAK values into the model, which statistically implies an association between tobacco and sTWEAK. Therefore, sTWEAK could be a mechanism associated with the smoker's paradox, although it would need a deeper mechanistic study to confirm this association.

The weaknesses of our work are obvious since it is a retrospective study that does not explore any mechanistic aspect. Experimental studies are necessary to confirm our conclusions. Another weakness is the analysis of the sTWEAK instead of the TWEAK-Fn14 association.

In conclusion, sTWEAK was associated with a good functional outcome in smoker patients with stroke undergoing reperfusion therapy.

## Data Availability Statement

The original contributions presented in the study are included in the article/Supplementary Material, further inquiries can be directed to the corresponding authors.

## Ethics Statement

The studies involving human participants were reviewed and approved by Clinical Research Ethics Committee of Galicia (registration codes 2019/616 and 2016/399). The patients/participants provided their written informed consent to participate in this study.

## Author Contributions

RI-R, JC, and PH: design and conceptualized the study, analyzed the data, and wrote the manuscript. IL-D, MR-Y, JP, AC, MA-A, AS-C, TS, and FC: major role in the acquisition of data and critical revision of the manuscript. All authors contributed to the article and approved the submitted version.

## Funding

This study was partially supported by grants from the Spanish Ministry of Science and Innovation (SAF2017-84267-R; PDC2021-121455-100), the Xunta de Galicia (Axencia Galega de Innovación-GAIN; Consellería de Cultura, Educación e Universidade: IN607A2018/3), the Instituto de Salud Carlos III (ISCIII) (PI17/00540; PI17/01103; PI20/01014; PI21/01256; AC21_2/00014; RETICS-INVICTUS PLUS - RD16/0019/0001; RICORS ICTUS - RD21/0006/0003), co funded by European Union, FEDER program. Furthermore, TS (CPII17/00027) and FC (CPII19/00020) are recipients of research contracts from the Miguel Servet Program of Instituto de Salud Carlos III. AS-C (CD20/00054) recipient of Sara Borrell grant funded by Instituto de Salud Carlos III and co-funded by European Union. The sponsors did not participate in the study design, collection, analysis, or interpretation of the data, in writing the report, or in the decision to submit the paper for publication.

## Conflict of Interest

The authors declare that the research was conducted in the absence of any commercial or financial relationships that could be construed as a potential conflict of interest.

## Publisher's Note

All claims expressed in this article are solely those of the authors and do not necessarily represent those of their affiliated organizations, or those of the publisher, the editors and the reviewers. Any product that may be evaluated in this article, or claim that may be made by its manufacturer, is not guaranteed or endorsed by the publisher.
