# Supplementary material for: The Smoking Paradox in Stroke Patients Under Reperfusion Treatment Is Associated With Endothelial Dysfunction
Source: Front Neurol. 2022 Mar 24;13:841484. doi: 10.3389/fneur.2022.841484 (PMC8987913; doi:10.3389/fneur.2022.841484)
Supplement: Supplementary file 1 [file Table_1.DOCX]

**Supplemental information**

**TABLE S1.** Clinical variables, biochemical parameters and neuroimaging values of patients classified according to the functional outcome at 3 months.

|  | Good outcome  n = 606 | Poor outcome  n = 269 | p |
| --- | --- | --- | --- |
| Age, years | 71.1 ± 12.6 | 74.1 ± 12.1 | 0.001 |
| Woman, % | 42.2 | 54.3 | 0.001 |
| Onset-treatment time, min | 154.6 ± 60.0 | 178.2 ± 60.8 | <0.0001 |
| Previous mRS | 0 [0, 0] | 0 [0, 0] | 0.145 |
| Arterial hypertension, % | 63.4 | 64.7 | 0.145 |
| Diabetes, % | 22.3 | 24.2 | 0.542 |
| Smoker, % | 25.0 | 16.2 | <0.0001 |
| Alcohol abuse, % | 10.6 | 9.7 | 0.719 |
| Dyslipidemia, % | 39.1 | 39.8 | 0.881 |
| Peripheral arterial disease, % | 6.4 | 7.4 | 0.563 |
| Atrial fibrillation, % | 21.5 | 26.0 | 0.139 |
| Ischemic heart disease, % | 12.0 | 14.1 | 0.441 |
| Heart failure, % | 4.3 | 4.5 | 0.918 |
| Axillary temperature, ºC | 36.2 ± 0.6 | 36.7 ± 0.8 | <0.0001 |
| Blood glucose, mg/dL | 133.3 ± 53.3 | 150.7 ± 58.9 | <0.0001 |
| Leucocytes x 10^3^/mL | 7.9 ± 2.9 | 9.3 ± 3.5 | <0.0001 |
| Fibrinogen, mg/dL | 403.1 ± 102.2 | 439.2 ± 101.3 | <0.0001 |
| C-reactive protein, mg/L | 3.2 ± 3.9 | 6.0 ± 4.5 | <0.0001 |
| Glycosylated hemoglobin, % | 6.3 ± 5.3 | 6.1 ± 1.3 | 0.435 |
| LDL-cholesterol, mg/dL | 108.5 ± 41.8 | 106.8 ± 38.1 | 0.648 |
| HDL-cholesterol, mg/dL | 41.1 ± 15.0 | 42.9 ± 14.7 | 0.199 |
| Triglycerides, mg/dL | 112.6 ± 48.2 | 117.6 ± 57.9 | 0.247 |
| NIHSS at admission | 16 [12, 20] | 19 [15, 23] | <0.0001 |
| TOAST |  |  | 0.068 |
| Atherothrombotic, % | 24.0 | 22.3 |  |
| Cardioembolic, % | 40.1 | 52.2 |  |
| Lacunar, % | 1.8 | - |  |
| Undetermined, % | 34.1 | 25.4 |  |
| DWI volume at admission, mL | 23.1 ± 43.2 | 43.7 ± 41.0 | 0.001 |
| CT volume 4th-7th day, mL | 24.6 ± 40.0 | 110.8 ± 99.8 | <0.0001 |
| Leukoaraiosis, % | 34.9 | 90.7 | <0.0001 |
| Degree of leukoaraiosis |  |  | <0.0001 |
| No, % | 16.7 | 0.9 |  |
|  |  |  |  |
| Fazecas I, % | 61.1 | 11.9 |  |
| Fazecas II, % | 22.2 | 25.7 |  |
| Fazecas III, % | - | 61.5 |  |
| Hemorrhagic transformation, % |  |  | <0.0001 |
| No, % | 67.7 | 68.4 |  |
| IH1, % | 26.5 | 10.8 |  |
| IH2, % | 4.5 | 7.1 |  |
| PH1, % | 1.0 | 7.4 |  |
| PH2, % | 0.7 | 6.3 |  |
| Neurological improvement $\geq$ 8, % | 58.6 | 11.2 | <0.0001 |
| Early neurological deterioration, % | 2.1 | 28.8 | <0.0001 |
| mRS at discharge | 2 [1, 3] | 5 [4, 6] | <0.0001 |
| Recanalization method |  |  |  |
| Thrombolysis | 71.7 | 28.3 | 0.0001 |
| Thrombectomy | 70.1 | 29.9 | 0.0001 |
| Thrombolysis followed by thrombectomy | 74.4 | 25.6 | 0.0001 |
| sTWEAK (pg/mL) | 3405 [2329 - 6629] | 10284 [7388-132478] | <0.0001 |

**TABLE 2S** Logistic regression models for factors associated with smoking habit.

| Independent variables | Not adjusted | | | Adjusted | | | |  |
| --- | --- | --- | --- | --- | --- | --- | --- | --- |
|  | OR | CI 95% | p | | OR | CI 95% | p | |
| Age | 0.97 | 0.95 - 0.98 | <0.0001 | | 0.97 | 0.96 - 0.99 | <0.0001 | |
| Women | 0.53 | 0.38 - 0.73 | <0.0001 | | 0.73 | 0.51 - 1.05 | 0.092 | |
| Hypertension | 0.63 | 0.46 - 0.87 | 0.001 | | 0.93 | 0.64 - 1.34 | 0.685 | |
| Diabetes | 0.62 | 0.41 - 0.93 | 0.021 | | 0.69 | 0.44 - 1.07 | 0.097 | |
| Alcohol | 3.95 | 2.52 - 6.14 | <0.0001 | | 3.27 | 2.03 - 5.27 | <0.0001 | |
| Atrial fibrillation | 0.46 | 0.30 - 0.72 | 0.001 | | 0.66 | 0.41 - 1.05 | 0.078 | |
| Fibrinogen | 0.99 | 0.99 - 0.99 | 0.002 | | 0.99 | 0.99 - 0.99 | 0.009 | |
| Leukoaraiosis | 0.69 | 0.50 - 0.95 | 0.024 | | 0.79 | 0.56 - 1.12 | 0.190 | |
